# Responding to and managing multijurisdictional outbreaks of COVID-19 in Canadian industrial worksite/work camp settings

**DOI:** 10.17269/s41997-024-00887-5

**Published:** 2024-04-29

**Authors:** Erin McGill, Anna Bellos, Andrea Nwosu, Adrian Zetner, Andrea Tyler, Natalie Knox, Kristyn Franklin, Kaitlin Patterson

**Affiliations:** 1 Infectious Disease Programs Branch, Public Health Agency of Canada, Ottawa, ON Canada; 2 National Microbiology Laboratory, Public Health Agency of Canada, Winnipeg, MB Canada; 3 Infectious Disease Programs Branch, Public Health Agency of Canada, Moncton, NB Canada

**Keywords:** COVID-19, Outbreak, Surveillance, Public health, Infectious disease, COVID-19, éclosion, surveillance, santé publique, maladie infectieuse

## Abstract

**Setting:**

Early in the COVID-19 pandemic, the Public Health Agency of Canada (PHAC) and provincial/territorial (P/T) public health identified the need for a coordinated response to complex multijurisdictional COVID-19 outbreaks. The first large multijurisdictional industrial worksite COVID-19 outbreak highlighted the risk of transmission within these congregate work settings, the risk of transmission to the broader community(ies), and the need to develop setting-specific outbreak response frameworks.

**Intervention:**

PHAC assembled a team to provide national outbreak support for multijurisdictional COVID-19 outbreaks in May 2020. The COVID-19 Outbreak Response Unit (ORU) worked with P/T partners to develop guiding principles for outbreak response and outbreak investigation processes, guidance documents, and investigation tools (e.g., minimum data elements and questionnaires).

**Outcomes:**

The ORU, P/T partners, and onsite industrial worksite health and safety staff leveraged outbreak investigation guidelines, industrial worksite outbreak process documents (including minimum data elements), and enhanced case questionnaires to respond to multiple COVID-19 outbreak investigations in industrial worksites. Clear roles/responsibilities and processes, along with standardized data, allowed for more efficient outbreak investigations and earlier implementation of mitigation measures.

**Implications:**

Multijurisdictional COVID-19 outbreaks highlighted the importance of public health collaboration with industry partners onsite. The assembly of a national outbreak response team was important to facilitate information sharing and provide technical support. Lessons learned and recommendations on outbreak preparation, detection, management, and communication are included to enhance a response framework applicable to future emerging or re-emerging pathogens with epidemic and/or pandemic potential.

**Supplementary information:**

The online version contains supplementary material available at 10.17269/s41997-024-00887-5.

## Résumé

**Contexte:**

Au début de la pandémie de COVID-19, l’Agence de la santé publique du Canada (ASPC) et les autorités provinciales/territoriales de santé publique ont reconnu la nécessité d’une réponse coordonnée en cas d’éclosions complexes multi-juridictionnelles de COVID-19. La première grande éclosion multi-juridictionnelle de COVID-19 dans un chantier industriel a mis en évidence le risque de transmission dans ces milieux de travail collectifs, le risque de transmission à l’ensemble de la (des) communauté(s) et la nécessité d’élaborer des cadres d’intervention en cas d’éclosion spécifiques aux types de milieux.

**Intervention:**

L’ASPC a formé une équipe chargée de soutenir la réponse nationale contre les éclosions multi-juridictionnelles de COVID-19 en mai 2020. L’Unité d’intervention en cas d’éclosion (UIE) de COVID-19 a collaboré avec des partenaires provinciaux et territoriaux pour élaborer des principes de référence pour la lutte contre les éclosions de COVID-19 et des processus d’enquête sur les éclosions, des documents d’orientation et des outils d’enquête (p.ex. des éléments de données minimales et des questionnaires).

**Résultats:**

L’UIE, les provinces et territoires et le personnel chargé de la santé et sécurité du travail sur le site se sont appuyés sur des principes de référence aux enquêtes sur les éclosions, les documents de processus d’enquête sur les éclosions dans les sites industriels, y compris les éléments de données minimales et le questionnaire détaillé sur les cas, pour répondre à multiples enquêtes d’éclosions de COVID-19 dans les sites industriels. Des rôles/responsabilités et des processus clairs, ainsi que des données standardisées, ont permis de mener des enquêtes plus efficaces sur les éclosions et de mettre en œuvre plus rapidement des mesures d’atténuation.

**Implications:**

Les éclosions multi-juridictionnelles de COVID-19 ont mis en évidence l’importance de la collaboration entre les autorités de santé publique et les partenaires industriels sur site. La constitution d’une équipe nationale d’intervention en cas d’éclosion a été importante pour faciliter le partage des informations et fournir un soutien technique. Les connaissances acquises et les recommandations sur la préparation, la détection, la gestion et la communication des éclosions sont incluses afin d’améliorer le cadre de réponse aux futurs agents pathogènes émergents ou ré-émergents ayant un potentiel épidémique et/ou pandémique.

## Setting

The World Health Organization (WHO) declared COVID-19 a pandemic on March 11, 2020 (WHO, 2020). Early in the pandemic, there was a paucity of evidence on transmission, high-risk settings, and the most effective public health interventions for COVID-19. Furthermore, limited testing was available; polymerase chain reaction (PCR) tests, while highly accurate, required specialized laboratory equipment and took 2–7 days to get results depending on the location and setting of testing (PHO, 2023).

There have been many industrial worksite/work camp (e.g., oil sands, diamond mines, potash mines, treatment facilities) COVID-19 outbreaks identified in Canada since the start of the pandemic (Dam et al., 2023). Thousands of Canadians commute from their province or territory (P/T) of residence to another P/T for rotational work. Out-of-province/territorial (OOP) rotational work typically requires workers to live and work in congregate settings, and some work at multiple worksites. As workers rotate in and out of their jurisdictions of residence, they are not only at higher risk of getting COVID-19 but can also act as vehicles for the introduction and spread of the virus within and between Canadian jurisdictions (Cooke, 2020; Nunn et al., 2023).

Canada is a federal state and the responsibility for delivering health services, including public health services, belongs to provincial and territorial governments. The Public Health Agency of Canada (PHAC) is part of the federal health portfolio and receives data from P/T health authorities. In the event of an outbreak, provincial and local health authorities are the lead investigators and work with their respective P/T partners (e.g., P/T Ministries of Labour and/or occupational health as appropriate) and industry. PHAC does provide outbreak support and resources at the request of P/Ts and a leadership role in multijurisdictional outbreaks.

Early detection and notification of an outbreak with the potential for interjurisdictional spread is essential to an effective multijurisdictional outbreak response (CDC, 2018). Delays in outbreak notifications in industrial settings have resulted in the spread of outbreaks to multiple jurisdictions (Nunn et al., 2023). There are numerous examples of rotational workers returning from industrial worksites with active outbreaks spreading SARS-CoV-2 to household members and starting community outbreaks in their home jurisdiction (Cooke, 2020; Tuttle, 2021; Reuters, 2020; Energy Mix, 2020).

The first large multijurisdictional industrial worksite COVID-19 outbreaks in Canada identified in early 2020 highlighted the risk of transmission within these congregate work settings and the challenges associated with managing complex multijurisdictional outbreaks. In Canada, public health responsibilities are divided among federal, P/T, and local/regional authorities, with outbreak detection and management falling under the jurisdiction where the outbreak is located. In early 2020, there was no federal group with the mandate to coordinate and respond to multijurisdictional COVID-19 outbreaks, and processes and tools to respond to complex COVID-19 outbreaks affecting multiple jurisdictions had not yet been established.

The purpose of the following communication is to describe PHAC’s approach to responding to and managing multijurisdictional COVID-19 outbreaks in industrial worksite/work camp settings and outline the challenges faced and lessons learned. The lessons learned and the framework for responding to complex multijurisdictional respiratory outbreaks can help inform the response to outbreaks of emerging or re-emerging pathogens with epidemic and/or pandemic potential.

## Intervention

PHAC rapidly leveraged the Foodborne Illness Outbreak Response Protocol (FIORP) (PHAC, 2017), with some adaptations to respond to the first large multijurisdictional COVID-19 outbreak. Although the FIORP provided excellent guidance for multijurisdictional investigations, there are key differences when managing outbreaks caused by a familiar enteric pathogen versus a novel respiratory pathogen (e.g., partners, mode of transmission, outbreak investigation methods, control practices). Adaptations were required in terms of the partners invited to the outbreak investigation calls, information collected from cases, type of analyses, and outbreak management strategies.

The need for federal outbreak support to respond to multijurisdictional COVID-19 outbreaks was identified. In order to address this need, PHAC assembled the COVID-19 Outbreak Response Unit (ORU) in May 2020. Individuals with outbreak investigation experience across PHAC and external to PHAC were recruited to join the ORU with the goal of modeling the team after the existing foodborne outbreak team, the Outbreak Management Division. The ORU team rapidly expanded over the summer of 2020 to include individuals with subject matter expertise, medical experience, data management, and analytic experience. The ORU was created with the mandate to support public health partners in their outbreak response by leading the development of outbreak management and investigation resources and procedures, and mobilizing staff to provide technical support (e.g., epidemiological analyses) for the outbreak investigation. The establishment of this unit was novel; previously PHAC has only had permanent outbreak response for foodborne outbreaks. Historically, public health emergencies (including non-foodborne outbreaks) would have used emergency operations centre management structures which are temporary.

The COVID-19 ORU addressed many of the challenges identified during the early multijurisdictional COVID-19 outbreaks in industrial worksite/work camp settings in Canada. The collaboration between industry partners onsite (i.e., occupational health and safety) and public health partners facilitated the management of complex outbreaks.

## Outcome

The ORU worked with P/T public health partners to develop processes for managing multijurisdictional/complex COVID-19 outbreaks. This included creating a process document for industrial worksite outbreaks that outlined the minimum data elements to share with partners during an outbreak, and the flow of information sharing. These processes were not finalized until May 2022; however, drafts of these process documents were in use by June 2020. The collaborative effort to develop outbreak protocols was useful in clarifying the roles and responsibilities of partners to improve outbreak management and response.

Evidence emerged and evolved over the course of the pandemic—evidence of airborne transmission emerged, testing and treatment strategies changed, vaccines were introduced, and the circulating virus evolved (Katella, 2023; Lewis, 2022; Edjoc et al., 2020; PHAC, 2023). Throughout this, the ORU worked with public health partners to establish and update outbreak, case, and contact definitions based on the best available evidence. These definitions were applied in other industrial worksite outbreak contexts, for example, oil sands, potash mine, and diamond mine.

The ORU developed and implemented enhanced setting-specific questionnaires to systematically collect the information necessary for outbreak response. This allowed information specific to industrial cases and contacts to be consistently collected to supplement the information collected in the national COVID-19 case report form (Supplementary Material, Appendix 1). The supplemental information was extremely useful in understanding transmission at the worksite (e.g., work rotation dates and work group details). The ORU, in collaboration with public health partners, also developed a list of minimum case data elements to be shared between jurisdictions (Supplementary Material, Appendix 2). The minimum data element list allowed for consistent information sharing between jurisdictions.

In 2021, the ORU published the COVID-19 Outbreak Investigation Toolkit in the PHAC training portal for public health professionals (COVID-19 Outbreak Investigation Toolkit, 2021). The Toolkit provided resources that public health professionals could leverage in the event of an outbreak investigation.

### Process improvements

The Outbreak Investigation Coordinating Committee (OICC) involves several organizations at various levels of government to facilitate information sharing in response to an outbreak involving cases from more than one P/T. PHAC coordinates the OICC and includes P/Ts with cases and relevant industry partners onsite.

Following the guidance outlined in the process documents, the ORU facilitated information sharing through OICC calls and transferred case/contact line lists between jurisdictions. A coordinated information sharing approach with clear roles and responsibilities was helpful during these complex multijurisdictional outbreaks. P/T regional health authorities indicated it was useful to clarify at the start of the investigation what the roles and responsibilities were, along with any specific outbreak investigation objectives (e.g., understanding transmission dynamics, mitigating further transmission, P/T situational updates).

The Canadian Network for Public Health Intelligence (CNPHI) is an online platform with the goal of fostering collaboration and consultation for surveillance, research, and outbreak response to support public health. To ensure timely notification of outbreaks with the potential for multijurisdictional spread, CNPHI was leveraged to distribute Public Health Alerts (PHAs). PHAC encouraged P/Ts to post these alerts via CNPHI if an industrial worksite outbreak with OOP workers was detected. These PHAs were used to notify public health partners of potential multijurisdictional outbreaks and would contain information on which P/Ts had workers present onsite, number of cases identified to date, and other pertinent details. For timely notification, PHAC and P/Ts agreed that posting an initial PHA within 72 h of detecting an outbreak with the potential for multijurisdictional spread being declared was crucial.

Resources developed (e.g., enhanced case questionnaire and minimum data elements) were an iterative process and could be modified to suit the specific outbreak being investigated. These internal documents were eventually made publicly available as resources for other public health professionals investigating SARS-CoV-2 outbreaks.

**Fig. 1:**
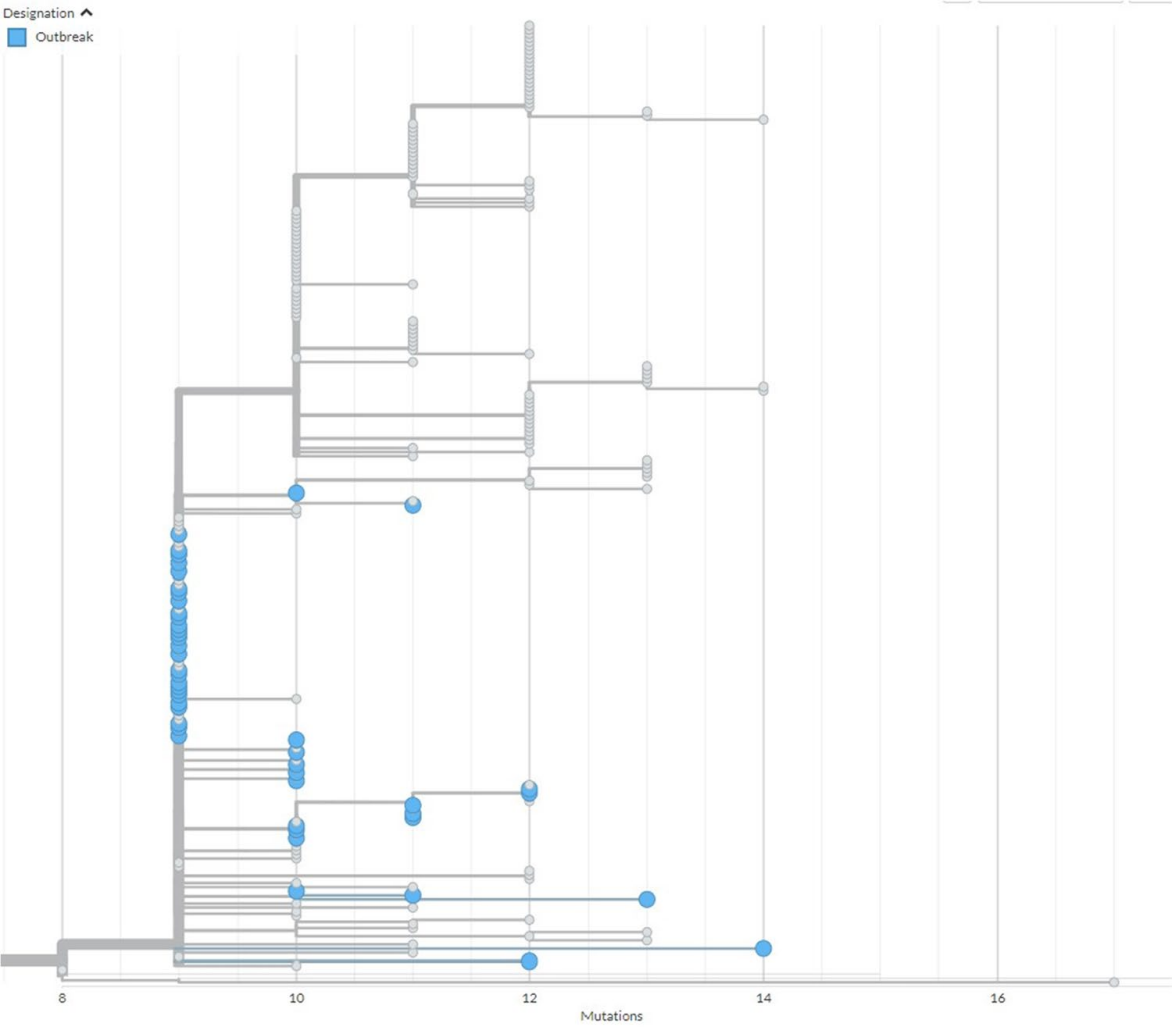
Samples from one outbreak are presented here in the context of concurrently collected surveillance sequences. The *x*-axis represents divergence (mutations) from the root sequence of a larger tree from which this clade has been excised. Little genetic diversity (0–3 mutations) is seen among outbreak sequences with the number of mutations over the time period found to be in line with estimated molecular clock (2–3 mutations per month) of wild-type SARS-CoV-2. This limited diversity among outbreak sequences may indicate a single introduction and the subsequent spread of virus

The utility of linking genomic and epidemiological information together to create a more complete picture was demonstrated during several outbreak investigations the ORU supported, where isolates were shared with the National Microbiology Laboratory for sequencing. The role of genomics in respiratory outbreak investigations at the national level was a new approach with many lessons learned which can be used for future respiratory outbreak investigations. Genomics has been established as very useful during foodborne outbreak investigations; however, using it to aid in near real-time outbreak investigations for rapidly spreading respiratory pathogens, including COVID-19, was novel (Diplock, 2022). This work adds to the body of evidence demonstrating the utility of genomics in contributing to outbreak response across a variety of settings and applications. Genomics and phylogenetic trees were used to link cases based on genetic relatedness and the epidemiological details were used to understand how the connections most likely occurred (e.g., the same work group or rotation). Genomics has shed light on the initial and/or multiple introductions of SARS-CoV-2 into work camps and subsequent introductions into communities linked to worksite outbreaks. Figure 1 demonstrates the findings from combined genomic and epidemiologic analyses that show that multiple outbreak cases sparked chains of transmission outside of the industrial workplace setting. Directionality of transmission in genomics has traditionally been challenging, but the large volume of genomic data generated from the COVID-19 pandemic made it possible to more accurately assess directionality.

### Challenges

Case and contact management evolved over the course of the pandemic as the scientific community’s understanding of COVID-19 changed. The extent to which case and contact management impacted outbreak investigations is not quantified. Workplace contacts were often not identified as close contacts because they did not meet the standard “close contact” definition, which focused on duration of time spent together and distance (i.e., greater than 15 min within 2 m), yet they often became cases. This reoccurring issue highlighted the importance of developing outbreak-specific case and contact definitions that consider the context of the worksite, and how outbreak investigations can be used to advance the understanding of transmission pathways of novel variants. Industrial worksites/work camps have similar transmission risk as congregate living facilities (e.g., communal living spaces, shared transportation, and/or shared break rooms). Additionally, the nature of industrial work often requires workers to be in close proximity. To address this, industrial worksite outbreak–specific definitions for cases and close contacts were drafted by ORU in collaboration with P/T public health partners.

Identifying contacts in industrial settings was further complicated by the intricacies of a complex workforce and the methods used by operators to track employees. The workforce is comprised of direct hires and employees of contracting and sub-contracting companies; there is often no centralized system to track all employees associated with a worksite. The lack of a centralized employee list and the continual movement of the work force on and off site continue to be unique challenges to contact tracing in these industrial sites.

OOP outbreak cases were often identified retrospectively. Employees would return to their jurisdiction of residence, develop symptoms and/or be identified as a close contact, get tested, and then be linked back to the worksite outbreak. This linking process often took days or weeks, preventing public health officials from addressing the outbreak at its source to reduce transmission. Adequate case and contact notification and management required substantial coordination between employers and public health authorities.

Another key challenge that arose during outbreak management was coordinating public health messaging between industry partners onsite and public health in multiple jurisdictions. Particularly in the first two years of the pandemic, provinces and territories had varying levels of public health measures and orders in place; employees returned to their home jurisdictions with mixed messaging on which measures applied to them and which to follow (e.g., varying isolation timelines). Instructions on public health measures can be clarified by worksites directing workers to follow the public health recommendations for their physical location, e.g., jurisdiction of residence or jurisdiction of workplace.

## Implications

In response to the challenges faced with multijurisdictional outbreaks, the ORU, in collaboration with P/T partners, developed several outbreak investigation tools, including the multijurisdictional industrial worksite outbreak process documents (including the minimum data elements to share between jurisdictions), an enhanced case questionnaire, and a document with the considerations for mass asymptomatic testing (COVID-19 Outbreak Investigation Toolkit, 2021). Additionally, during the pandemic’s first year, P/Ts often requested ORU support for industrial worksite/work camp outbreak investigations, particularly for information coordination, subject matter expertise, and analysis support. Feedback received from P/T regional health authorities during investigations, OICC, and pandemic working group meetings indicated that ORU support and tools were useful.

The outbreak processes and resources developed during the COVID-19 pandemic were informed by several complex multijurisdictional outbreak investigations. These can be adapted for future emerging or re-emerging pathogens with epidemic and/or pandemic potential. Here we provide critical success factors for managing multijurisdictional outbreaks in industrial/work camp settings in the context of a novel pathogen with widespread transmission in the community.

### National preparation

- Strong relationships between public health and occupational health and safety of industry partners onsite.
- Outbreak response protocols to identify the roles and responsibilities of all levels of public health partners and outline response activities for multijurisdictional outbreak investigations.
- Outbreak investigation tools (e.g., case notification process, questionnaires) to guide the investigation and management of multijurisdictional outbreaks.
- Inventory of individuals who can be mobilized and/or deployed with skillsets required for outbreak response (outbreak management, data management, analysis and understanding of the Canadian public health system).

### National outbreak detection and management

- Prompt reporting of suspected outbreaks in industrial worksite/work camp settings to local public health to facilitate rapid outbreak investigation and response.
- Close collaboration between on-site staff (e.g., occupational health and safety) and public health to develop case and contact definitions, and conduct case investigation and contact tracing.
- Develop, maintain, and update tools on the best available evidence to guide public health action for identifying cases and contacts in multijurisdictional outbreaks.
- Use both laboratory/genomic and epidemiological information to inform understanding of case dynamics and hypotheses regarding chains of transmission.

### Federal/provincial/territorial communication

- Timely notification to other jurisdictions regarding the detection of cases and/or the declaration of an outbreak linked to a setting where there is known risk of interjurisdictional spread (i.e., work settings with highly mobile population, employees who reside in other jurisdictions, or employees who reside in at-risk communities).
- Provide workers leaving a worksite/work camp with an ongoing outbreak with resources, including information on the outbreak and steps the individual should take once they leave the worksite, along with clear instructions on how to report and contact public health if they later test positive.

## Conclusion

PHAC’s COVID-19 Outbreak Response Unit worked with P/T regional public health authorities to develop guiding principles for outbreak response and outbreak investigation processes, guidance documents, and investigation tools (e.g., minimum data elements and questionnaires). These tools addressed common challenges caused by the complexities of managing multijurisdictional COVID-19 outbreaks in industrial worksite/work camp settings.

The ORU facilitated information sharing and provided technical support to multijurisdictional COVID-19 industrial outbreaks. A systematic approach with clear roles and responsibilities between workplace safety and public health in the context of a widespread community-level outbreak was important to mitigating further transmission. This work highlights the importance of public health collaboration with industry partners, including occupational health staff.

Tools created by the ORU in collaboration with public health partners to respond to multijurisdictional COVID-19 outbreaks can be modified to help manage future emerging or re-emerging pathogens with epidemic and/or pandemic potential in these complex settings.

## Implications for policy and practice

What are the innovations in this policy or program?

- Outbreak investigations that involve more than one jurisdiction can be complex, especially when responding to the threat of a novel pathogen. The Outbreak Response Unit (ORU) was assembled to provide federal outbreak support for multijurisdictional COVID-19 outbreaks. The ORU was novel because the team was assembled during a pandemic caused by an emerging pathogen. The team coordinated not only with provincial and territorial health authorities, but also with onsite industrial worksite health and safety staff.
- The creation of several outbreak investigation documents that required input from both federal and provincial/territorial public health authorities in a short time period was also unique as processes that implicate multiple jurisdictions usually take longer to coordinate and establish.

What are the burning research questions for this innovation?

- Multiple public health risks continue to emerge/re-emerge (e.g., avian influenza, mpox, measles), highlighting the importance of an outbreak response framework. Further work needs to be done to establish a federal response framework and ensure the guidance documents and tools are versatile to respond to threats of epidemic and/or pandemic potential.
- In order to support outbreak investigations, response planning for infectious diseases that are emerging or re-emerging should include a response framework, including guidance documents, tools, and the ability to rapidly assemble a team capable of supporting multijurisdictional and/or complex outbreaks.

### Supplementary information

Below is the link to the electronic supplementary material.

Supplementary file1 (PDF 317 KB)

Supplementary file2 (PDF 146 KB)

## Data Availability

Available upon request to the corresponding author.
