# Supplementary material for: Responding to and managing multijurisdictional outbreaks of COVID-19 in Canadian industrial worksite/work camp settings
Source: Can J Public Health. 2024 Apr 29;115(3):425–31. doi: 10.17269/s41997-024-00887-5 (PMC11133241; doi:10.17269/s41997-024-00887-5)
Supplement: Supplementary file 1 — Supplementary file1 (PDF 317 KB) [file 41997_2024_887_MOESM1_ESM.pdf]

Setting specific investigation considerations and methods

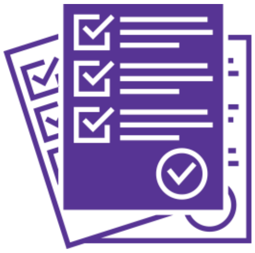

Industrial work site/work camp – case investigation questionnaire

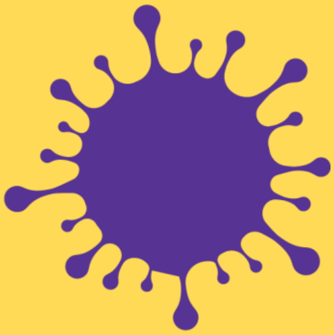

INSTRUCTIONS TO THE INVESTIGATOR / INTERVIEWER

The following is a case investigation questionnaire for an individual who is a case associated with an outbreak of COVID-19 at an industrial worksite/work camp. This questionnaire is intended to be implemented directly with the case or their proxy by the qualified interviewer. It should not be given to the case to complete on their own.

The case investigation interview may be a stressful event for the interviewee and efforts should be made to conduct the interview in a non-judgemental manner to avoid stigmatization and ensure the interviewee is as comfortable as possible. Throughout the interview, the interviewer should take into consideration the interviewee's socio-demographic situation (gender, race, Indigeneity, and/or first language spoken).

Important information to assist with the investigation include:

1. Map of work site, meal locations, communal settings and on-site accommodations
2. Roster of all workers, with contact and accommodation information (with some sort of unique ID)
3. Existing environmental health inspection information of site (and maintain any inspection results and information going forward)

Last updated:
6 August 2021

Public Health Agency of Canada
Agence de la santé publique du Canada

Canada

ADMINISTRATIVE INFORMATION

This information should be collected by the investigator prior to interviewing the case.

Interviewer's first name:

Interviewer's last name:

Interviewer's telephone number:

Email Address:

Interviewer's designation:

Date of Interview:

Investigation ID:

Outbreak ID:

Outbreak/investigation name/setting:

Location / Address:

Outbreak/investigation lead(s):

Primary contact at worksite:

Primary contact's phone number: **Email:**

Outbreak start date:

Outbreak end date:

Date outbreak declared:

Date outbreak resolved (if resolved):

Environmental inspection

- Date of inspection of last workplace inspection:
- Inspectors:
  - Health Unit
  - Ministry of Labour
  - Other agency:
- Was the site compliant:
  - Yes
  - No
  - Cannot assess
  - If no, describe issues:

Total number of employees/workers:

Total number of communal sites (living and sleeping locations):

Contact with EMS/first responders on site?

- Yes
- No
- Unknown

Contact with mobile health assessment team?

- Yes
- No
- Unknown

Other agencies involved in response:

- Red Cross
- Provincial Ministry of Labour
- Provincial Health
- Other (specify):

CASE INFORMATION

To be collected and recorded by the investigator prior to the case interview.

Case ID: **Public Health Unit:**

First name:

Last name:

Middle name:

Date of birth:

Case Status: ☐ **Confirmed** ☐ **Probable/ Epi-linked**

Symptomatic? ☐ **Yes** ☐ **No** ☐ **Unknown**

Symptom onset date (yyyy-mm-dd):

Specimen collection date (yyyy-mm-dd):

VOC Identified:

☐ Yes, specify name of VOC (e.g. B.1.1.7):

☐ No ☐ Unknown

INTRODUCTION

The following information is obtained via interview with the case. This information is collected for the purposes of the investigation and should be supplementary to the information collected by public health for case and contact management. Where possible, investigators should collect case information prior to the interview and confirm accuracy with the interviewee.

INTRODUCTION SCRIPT: A COVID-19 outbreak has been declared at _____________(work camp name) on _____________ (date(s)). and you have been identified as being onsite during the outbreak. Therefore, I would like to ask you a series of questions in order to help us better understand how you may have been exposed to the virus. This information will help to identify others that may have been exposed and help prevent further spread of the virus to other people.

CASE DETAILS

First name:

Last name:

Middle name:

Age: **Date of birth (yyyy-mm-dd):**

Gender:

☐ **Male** ☐ **Female** ☐ **Another gender**

Telephone number: **Email:**

Current address:

Is this questionnaire being completed by a proxy? ☐ **Yes** ☐ **No**

Proxy first name: **Proxy last name:**

Telephone: **Email:**

Proxy's relationship to the case:

SCRIPT: We know that people of different races do not have significantly different genetics. But our race still has important consequences, including how we are treated by different individuals and institutions.

Which race category best describes you? (select all that apply)

☐ Black (African, Afro-Caribbean, African Canadian descent)

☐ East Asian (Chinese, Korean, Japanese, Taiwanese descent)

☐ Southeast Asian (Filipino, Vietnamese, Cambodian, Thai, Indonesian, other Southeast Asian descent)

☐ Indigenous (First Nations, Inuk/Inuit, Métis)

☐ Yes, First Nations ☐ Yes, Métis ☐ Yes, Inuk/Inuit ☐ No
☐ Prefer not to answer

☐ Latinx (Latin American, Hispanic descent)

☐ Middle Eastern (Arab, Persian, West Asian descent (e.g., Afghan, Egyptian, Iranian, Lebanese, Turkish, Kurdish))

- [ ] South Asian (South Asian descent (e.g., East Indian, Pakistani, Bangladeshi, Sri Lankan, Indo-Caribbean))
- [ ] White (European descent)
- [ ] Another Race Category (specify): ____
- [ ] Do not know
- [ ] Prefer not to answer

SCRIPT: We would like to understand your level of protection against COVID-19.

Have you received your COVID-19 vaccination?

- [ ] Yes

  COVID-19 First dose: ____ (yyyy-mm-dd)
  Product: ____
  COVID-19 Second dose: ____ (yyyy-mm-dd)
  Product: ____
  COVID-19 Third dose: ____ (yyyy-mm-dd)
  Product: ____

- [ ] No
- [ ] Prefer not to answer

Have you been previously infected with COVID-19?

- [ ] Yes

  If yes, were you symptomatic? [ ] Yes [ ] No [ ] Unknown

  Date of symptom onset: (yyyy-mm-dd) ____

  If yes, date of positive test: (yyyy-mm-dd) ____

- [ ] No
- [ ] Do not know

CLINICAL COURSE AND OUTCOMES

In the 14 days prior to your departure date, did you develop any of the following symptoms? If yes, when did the symptom(s) first start (yyyy-mm-dd)?

- [ ] new or worsening cough date: ____
- [ ] shortness of breath or difficulty breathing. date: ____

- [ ] temperature equal to or over 38°C. date:
- [ ] feeling feverish. date:
- [ ] chills date:
- [ ] fatigue or weakness date:
- [ ] muscle or body aches date:
- [ ] new loss of smell or taste date:
- [ ] headache date:
- [ ] gastrointestinal symptoms (abdominal pain, diarrhea, vomiting) date:
- [ ] feeling very unwell date:
- [ ] other date:
- [ ] no symptoms

Referred to a Mobile Health Assessment Team ☐ Yes ☐ No ☐ Unknown

Referred to Hospital/EMS ☐ Yes ☐ No ☐ Unknown

Isolation location

- [ ] Hotel
- [ ] Group living
- [ ] Private residence
- [ ] Other (specify):

Isolation Unit

Isolation Start date: **Isolation end date:**

If you became ill, were you admitted to the hospital?

- [ ] Yes
  - Admission date (yyyy-mm-dd)
  - Discharge date (yyyy-mm-dd)
- [ ] No
- [ ] Unknown

Were you admitted to the intensive care unit (ICU)

- [ ] Yes
  - Admission date (yyyy-mm-dd)
  - Discharge date (yyyy-mm-dd)
- [ ] No

Case deceased

- [ ] Yes
  - Date of death (yyyy-mm-dd)
- [ ] No

WORK DETAILS

Prior to the onset of your symptoms on ________________________ [onset date] OR ________________________ [test date] if you were identified as an asymptomatic case:

When was the work rotation at the ______________________________ [work camp name] during which you experienced onset of symptoms of COVID-19 or were identified as an asymptomatic COVID-19 case?

Start date (yyy-mm-dd):

End date (yyy-mm-dd):

For the next series of questions, please answer to the best of your ability based on the your most recent work rotation between (yyyy-mm-dd) and (yyyy-mm-dd)

Please provide your next scheduled rotation date.

Start date (yyy-mm-dd):

End date (yyy-mm-dd):

Last day of work before isolation:

First day of return to work:

Type of employment?

- Employee (specify name of employer):
- Contractor (specify agency/employer):
- Other (describe):

Workplace(s):

Area(s) of work (where employee typically spends work day):

Please select the option that best describes your role/job during your rotation:

- Mining
- Power house
- Maintenance facilities
- Secondary extraction
- Bitumen processing
- Ore preparation plant
- Control rooms
- Primary extraction and tailings
- Froth processing
- Construction

- [ ] Cleaning/Housekeeping
- [ ] Cook/Kitchen
- [ ] Firefighter/First Responder
- [ ] Deliveries
- [ ] Other, specify:

If Other, or if the above, does not capture your role/job please provide your job title and any additional details:

Please select the option that best describes where you spent most of your work day on your rotation:

- [ ] Mining
- [ ] Bitumen Production
- [ ] Upgrading & Utilities
- [ ] Facilities & Services
- [ ] Major Projects
- [ ] Supply Management
- [ ] Lodging
- [ ] Dining Services
- [ ] Other, specify:

Please provide any additional description of where on site you worked during your rotation.

Please circle on the map provided any area(s) that you spent more than 25% of your work day.

Please list your work group(s) name during your rotation.

- [ ] 1st Shift:
- [ ] 2nd Shift:
- [ ] 3rd Shift:
- [ ] 4th Shift:

Please list your work group(s) name during your rotation.

- [ ] 4/3 Rotation:
- [ ] 7/7 Rotation:
- [ ] 8/6 Rotation:
- [ ] 14/14 Rotation:
- [ ] Other, specify:

LODGING AND MEALS

While on rotation do you live: ☐ On site ☐ Off site ☐ Did not require lodging

Please list the name of your accommodation, floor, wing, room, check-in and checkout dates while you were on your rotation.

#1:

Accommodation Name:

Address:

Floor: Room#:

Check-in date (yyyy-mm-dd):

Check-out date (yyyy-mm-dd):

#2:

Accommodation Name:

Address:

Floor:

Room#:

Check-in date (yyyy-mm-dd):

Check-out date (yyyy-mm-dd):

#3:

Accommodation Name:

Address:

Floor:

Room#:

Check-in date (yyyy-mm-dd):

Check-out date (yyyy-mm-dd):

Please describe where and when do you generally pick up meals during your rotation

☐ Breakfast:

☐ Lunch:

☐ Dinner:

TRANSPORTATION

Please describe any off-site ground transportation you used to go from your accommodation to worksite or between site locations during your rotation.

Transport #1:

Date(s) for which this transport detail applies (yyyy-mm-dd):

Mode of transport: ☐ Private vehicle ☐ Public transport ☐ Taxi ☐ Ride-share

☐ Other (provide details):

Route name (if applicable):

Route start point:

Route endpoint:

Names of fellow travellers/passengers (if known):

Other details:

Transport #2:

Date(s) for which this transport detail applies (yyyy-mm-dd):

Mode of transport: ☐ Private vehicle ☐ Public transport ☐ Taxi ☐ Ride-share

☐ Other (provide details):

Route name (if applicable):

Route start point:

Route endpoint:

Names of fellow travellers/passengers (if known):

Other details:

Transport #3:

Date(s) for which this transport detail applies (yyyy-mm-dd):

Mode of transport: ☐ Private vehicle ☐ Public transport ☐ Taxi ☐ Ride-share

☐ Other (provide details):

Route name (if applicable):

Route start point:

Route endpoint:

Names of fellow travellers/passengers (if known):

Other details:

How did you make your way from your home city/province to the worksite city/province? Please select all that apply.

- [ ] Flight
- [ ] Ground Transportation
- [ ] Other, specify:

If you took an airplane for any part of your in-bound travel (i.e. from your home city/province to the worksite city/province), please provide details for each flight.

#1:

- [ ] Commercial flight
- [ ] Charter flight

Origin:

Destination:

Flight date (yyyy-mm-dd): Flight time (HH:MM):

Airline/flight number): Seat number:

Alone or with others:

#2:

- [ ] Commercial flight
- [ ] Charter flight

Origin:

Destination:

Flight date (yyyy-mm-dd): Flight time (HH:MM):

Airline/flight number): Seat number:

Alone or with others:

#3:

- [ ] Commercial flight
- [ ] Charter flight

Origin:

Destination:

Flight date (yyyy-mm-dd): Flight time (HH:MM):

Airline/flight number): Seat number:

Alone or with others:

If you took ground transportation for any part of your in-bound travel (i.e. from your home city/province to the worksite city/province) please provide details for each leg of your travel.

Transport #1:

Date(s) for which this transport detail applies (yyyy-mm-dd):

Mode of transport: ☐ Private vehicle ☐ Public transport ☐ Taxi ☐ Ride-share

☐ Other (provide details):

Route name (if applicable):

Route start point:

Route endpoint:

Names of fellow travellers/passengers (if known):

Other details:

How did you make your way from the worksite back to your home city/province? Please select all that apply

☐ Flight: ☐ Ground Transportation

☐ Other, specify:

If you took an airplane for any part of your out-bound travel (i.e. from the worksite city/province to your home city/province) please provide details for each flight.

#1:

☐ Commercial flight ☐ Charter flight

Origin:

Destination:

Travel date (yyyy-mm-dd): Flight time (HH:MM):

Airline/flight number): Seat number:

Alone or with others:

#2:

☐ Commercial flight ☐ Charter flight

Origin:

Destination:

Travel date (yyyy-mm-dd): Flight time (HH:MM):

Airline/flight number): Seat number:

Alone or with others:

#3:

Commercial flight Charter flight

Origin:

Destination:

Travel date (yyyy-mm-dd): Flight time (HH:MM):

Airline/flight number): Seat number:

Alone or with others:

If you took ground transportation for any part of your out-bound travel (i.e. from the worksite city/province to your home city/province) please provide details for each leg of your travel.

Transport #1:

Origin:

Destination:

Travel date (yyyy-mm-dd):

Mode (bus, drive, taxi, uber, other):

Alone or with others:

Detail:

Transport #2:

Origin:

Destination:

Travel date (yyyy-mm-dd):

Mode (bus, drive, taxi, uber, other):

Alone or with others:

Detail:

Transport #3:

Origin:

Destination:

Travel date (yyyy-mm-dd):

Mode (bus, drive, taxi, uber, other):

Alone or with others:

Detail:

EXPOSURES

SCRIPT: The following questions are about determining when and where you may have been exposed to COVID-19 in the 14 days prior to your onset of symptoms or the date of your test from (yyyy-mm-dd)______________________ to (yyyy-mm-dd)___________________________.

Contact with a confirmed or possible case:

During your most recent rotation, in the 14 days prior to your onset of symptoms or the date of your test did you have contact with someone who received or may have received a positive COVID-19 test, or who was or may have been diagnosed with COVID-19? The person (people) may not have been known to be positive at the time of the contact.

☐ Yes ☐ No ☐ Unknown

If Yes to previous question, provide the following details:

Case #1:

Name of case:

Date of contact:

Relationship:

Event/location of contact:

Frequency of contact:

Public health measures used (i.e. distancing, masking):

Case #2:

Name of case:

Date of contact:

Relationship:

Event/location of contact:

Frequency of contact:

Public health measures used (i.e. distancing, masking):

Case #3:

Name of case:

Date of contact:

Relationship:

Event/location of contact:

Frequency of contact:

Public health measures used (i.e. distancing, masking):

Case #4:

Name of case:

Date of contact:

Relationship:

Event/location of contact:

Frequency of contact:

Public health measures used: (i.e. distancing, masking):

Contact with a person with symptoms:

During your work rotation, in the 14 days prior to your onset of symptoms or the date of your test did you have contact with any individual(s) with symptoms (i.e. cough, shortness of breath or difficulty breathing, fever, chills, muscle pain, sore throat, new loss of taste or smell) who did not know they had COVID-19__________ [worksite name]?

Yes No Unknown

If Yes to previous question, please provide details of the contact if not already noted above:

Contact #1:

Name of person with symptoms:

Date of contact:

Relationship:

Event/location of contact:

Frequency of contact:

Public health measures used (i.e. distancing, masking?):

Contact #2:

Name of person with symptoms:

Date of contact:

Relationship:

Event/location of contact:

Frequency of contact:

Public health measures used (i.e. distancing, masking?):

Contact #3:

Name of person with symptoms:

Date of contact:

Relationship:

Event/location of contact:

Frequency of contact:

Public health measures used (i.e. distancing, masking?):

Contact #4:

Name of person with symptoms:

Date of contact:

Relationship:

Event/location of contact:

Frequency of contact:

Public health measures used (i.e. distancing, masking?):

During your work rotation, in the 14 days prior to your onset of symptoms or the date of your test (if you were asymptomatic) please provide the name(s) of any other common spaces where you came into close contact with others <u>you have not already described</u>.

Area #1:

Date:

Location:

Event/Activity:

Public health measures used:

Additional notes:

Area #2:

Date:

Location:

Event/Activity:

Public health measures used:

Additional notes:

Area #3:

Date:

Location:

Event/Activity:

Public health measures used:

Additional notes:

Area #4:

Date:

Location:

Event/Activity:

Public health measures used:

Additional notes:

CLOSE CONTACT INFORMATION

SCRIPT: The following questions are about places or people you may have been in contact with **while you were transmitting the virus***,

From (yyyy-mm-dd):____________________ to (yyyy-mm-dd):_________________________

*from 2 days prior to your onset of symptoms or the date of your test (if you were asymptomatic) until 10 days later or when you started isolation [Interviewer to provide the specific dates and use memory cues.]

Please provide the name(s) of ALL common and public spaces (e.g. workplace, lobby, cafeteria, car, shuttle bus, planes, and airport) or recreational activities where you came into close contact with others, which you have not already described.

Area #1:

Name:

Date:

Close contact location/details:

Public health measures used:

Area #2:

Name:

Date:

Close contact location/details:

Public health measures used:

Area #3:

Name:

Date:

Close contact location/details:

Public health measures used:

Area #4:

Name:

Date:

Close contact location/details:

Public health measures used:

Please provide the name(s) of ALL individuals in which you came into close contact, that were not already named above.

Contact #1:

Name:

Phone:

Location:

Relationship:

Date:

Public health measures used:

Contact #2:

Name:

Phone:

Location:

Relationship:

Date:

Public health used:

Contact #3:

Name:

Phone:

Location:

Relationship:

Date:

Public health measures used:

Contact #4:

Name:

Phone:
Location:
Relationship:
Date:
Public health measures used:

Are you aware of anyone else who you think we should talk to? This could be anyone who you think may have COVID-19 or who may have come in contact with someone who may have COVID-19.

Contact #1:

Name:

Phone:

Location:

Relationship:

Date:

Contact #2:

Name:

Phone:

Location:

Relationship:

Date:

Contact #3:

Name:

Phone:

Location:

Relationship:

Date:

Contact #4:

Name:

Phone:

Location:

Relationship:

Date:
