# Supplementary material for: Responding to and managing multijurisdictional outbreaks of COVID-19 in Canadian industrial worksite/work camp settings
Source: Can J Public Health. 2024 Apr 29;115(3):425–31. doi: 10.17269/s41997-024-00887-5 (PMC11133241; doi:10.17269/s41997-024-00887-5)
Supplement: Supplementary file 2 — Supplementary file2 (PDF 146 KB) [file 41997_2024_887_MOESM2_ESM.pdf]

Appendix II – Minimum list of data elements to share in the event of a multijurisdictional outbreak

Proposed variable list to share with PHAC and bilaterally between jurisdictions in the event of an industrial worksite/work camp outbreak investigation

Purpose	Variable class*	Variables*
**Reportable to PHAC for outbreak-specific case line list**	Primary cases and Secondary Cases (confirmed, probable)	P/T case ID
		Outbreak ID
		Reporting province/territory
		Province of residence
		Surveillance case classification (confirmed, probable)
		Symptom onset date
		Test result date
		Has a variant of concern, variant of interest or other mutation of interest been identified?
		Variant Screening Result
		Variant Sequencing Result
		Reported date
		Age
		Sex assigned at birth
		Gender
		Occupation information
		Exposure information
		Total number of contacts identified for this case
		Number of close contacts that became cases
		Admitted to the hospital as a result of their illness
		If hospitalized, was the case admitted to intensive care unit (ICU)
		If the case is deceased, was COVID-19 the cause of death or a contributing factor
		Did the case receive vaccination for COVID-19
		Indicate vaccine(s) received (list of names)
		Number of doses administered
		Date of first dose administered
		Date of second dose administered

**Information sharing between P/Ts for outbreak investigation**	Primary cases (confirmed, probable)	Full name
		DOB
		PHN
		Symptom onset date
		Specimen collection date
		Test result date
		Has a variant of concern, variant of interest or other mutation of interest been identified?
		Variant Screening Result
		Variant Sequencing Result
		Did the individual receive vaccination for COVID-19
		Indicate vaccine(s) received (list of names)
		Number of doses administered
		Date of first dose administered
		Date of second dose administered
		Full findings from case investigation
		Health region
		Address of residence
		Interprovincial travel
		Surveillance case classification (confirmed, probable)
		Date last at site of outbreak
		Employer name
		Work group
		Case epi-linked to another case
		Intraprovincial travel (i.e. within a P/T)
		Mode of travel
		Infectious during travel
		Return to work date
		Isolation date (start/end)
	Contact	Full name

		DOB
		PHN
		Specimen collection date
		Test result date
		Did the individual receive vaccination for COVID-19
		Indicate vaccine(s) received (list of names)
		Number of doses administered
		Date of first dose administered
		Date of second dose administered
		Phone number
		Health region
		Address of residence
		Name of source case
		Email
		Date last at site of outbreak
		Employer name
		Work group
		Last known contact with case
		Return to work date
		Quarantine date (start/end)

*Note: Information shared between P/Ts for the purpose of outbreak investigation is not to be shared publicly

**Essential variables are the minimum variables that need to be shared between P/Ts for the purpose of outbreak investigation; ideally the full list of variables here is shared between P/Ts if available.
